# Editorial: New trends in type 2 diabetes diagnosis and management in primary care

**DOI:** 10.3389/fmed.2023.1243319

**Published:** 2023-07-06

**Authors:** Aleksandra Klisic, I-Shiang Tzeng

**Affiliations:** ^1^Primary Health Care Center, University of Montenegro-Faculty of Medicine, Podgorica, Montenegro; ^2^Department of Statistics, School of Business, National Taipei University, New Taipei, Taiwan

**Keywords:** type 2 diabetes, primary care, general practice, family medicine, multimorbidity, insulin resistance

Type 2 diabetes mellitus (T2DM) is a global health concern (1–3), with rising prevalence especially in middle and low income countries (4). In order to raise awareness and to timely diagnose this metabolic disorder, as well as to prevent further complications especially in poor countries, it is of paramount importance to enable cost effective and easy available approaches for the diagnosis and management of T2DM (5, 6). The following editorial aims to provide an overview of the latest trends related to T2DM diagnosis and management in a primary care setting.

Chen et al. proposed easily obtained and beneficial parameter, i.e. white blood cell (WBC) count for the early detection of insulin resistance (IR) as measured by Homeostasis Model Assessment of Insulin Resistance (HOMA-IR) index in the elderly Taiwanese and alerted primary care physicians to pay attention to this population group concerning the increased risk of IR. Similarly, Han et al. in a large national survey 2011-2016 conducted in the United States that encompassed more than 5,000 participants showed a positive correlation between another cost-effective biomarker, i.e. serum uric acid and the risk for IR. Chocair et al. suggested lower cut-offs for insulin (8 mU/L in men and 10 mU/L in women) and the HOMA-IR index (1.5 in men and 2.0 in women) in Brazilians and proposed new classification for metabolic syndrome (MetS), as following: metabolically normal: cut-off insulin level as mentioned above and without the International Diabetes Federation (IDF) criteria for MetS diagnosis; Level 1 MetS: hyperinsulinemia, in addition to one or two IDF criteria for MetS diagnosis; Level 2 MetS: hyperinsulinemia, in addition to three or more IDF criteria for MetS diagnosis.

Pi et al. explored the utility of point-of-care (POC) capillary glycated hemoglobin A1c (HbA1c) in primary healthcare settings given the fact that rural Chinese settings are limited for more expensive standardized HbA1c measurement method. They found a strong positive correlation between POC capillary and venous HbA1c values. Moreover, POC for HbA1c demonstrated high discriminatory ability for identification of patients with abnormal glucose regulation and undiagnosed diabetes.

Wang et al. proposed magnetic resonance spectroscopy (MRS) as an early imaging diagnostic and prognostic assessment of stroke. They applied MRS, in addition to clinical neurological deficit score (NIHSS) and HbA1c values in 53 T2DM patients within 24 h after the acute ischemic stroke (AIS) onset. A positive correlation between HbA1c values and NIHSS in T2DM patients with AIS was shown. The MRS and clinical NIHSS score showed high consistency in evaluating AIS.

In addition to timely manner diagnosis, prompt and multifactorial treatment and intervention could postpone the onset of T2DM-related complications.

Lin et al. compared the effects of metformin-based dual therapy vs. triple therapy on changes of glycemic and lipid parameters in 60 Taiwanese T2DM patients who were given at least 24 months of metformin monotherapy, dual therapy, i.e. with sodium-glucose cotransporter-2 (SGLT2) inhibitors or dipeptidyl peptidase 4 (DPP4) inhibitors or triple therapy with metformin plus linagliptin (DPP-4 inhibitor) and dapagliflozin (SGLT2 inhibitor). The authors found similar ability of glycemic control with dual therapy with metformin and linagliptin, just like with triple therapy. Although being effective, the mentioned triple therapy could be costly, so the authors recommended dual therapy with metformin and linagliptin as the better solution for long-term glycemic control due to similar glucose control ability of mentioned dual and triple therapy.

Vlacho et al. conducted a national cross-sectional study among physicians from a large number of Spanish primary care centers in an attempt to examine the degree of adherence with the therapeutic recommendations of the Clinical Practice Guidelines among recently diagnosed T2DM individuals, frail and obese subjects. They recorded adequate adherence for the majority of examinees with the highest percentage in the recently diagnosed T2DM individuals. On the other hand, Fu et al. conducted the study that included more than 10,000 patients with long-lasting T2DM with a median follow-up of 8.8 years and found significantly higher risk of all-cause mortality in T2DM patients that live alone as compared with those living with one or more adults, thus presuming that individuals who live alone exhibit an increasing tendency toward poor health behaviors.

Jie et al. identified factors related to the activities of daily living (ADL) limitations in China, i.e., a sedentary lifestyle, difficulty in sleeping and suffering from stroke or malignant tumor. They showed that those factors may increase the risk of ADL limitations among older (≥70 years) patients with T2DM, suggesting that identification of such factors may add reliable information to the development of targeted nursing practice and the improvement of health management for older T2DM patients.

Sukhram et al. recorded a significant relationship between serum cotinine (i.e., the key metabolite of nicotine and an indicator of cigarette smoke which is highly related to cardiovascular disease onset) and lipid parameters and their indexes in T2DM patients. The authors suggested the need for the modification of mentioned behavioral risk factor in an attempt to prevent comorbidities and advance cardiovascular health outcomes.

Zhao et al. described as moderately good typical behavioral characteristics of patients associated with integrated treatment and prevention (ITP) services for T2DM in China. The duration of disease, health insurance and treatment modality independently predicted the patients' behaviors associated with ITP services for T2DM. The authors pointed out the need for the development and implementation targeted interventions for different groups with the goal of improving T2DM patients' behaviors associated with ITP services.

In an attempt to evaluate the providers' T2DM care quality in rural China, Wu et al. applied standardized patients (SPs) method as the “gold standard” for examination of the quality of clinical practice. They recorded poor quality of T2DM care in rural regions of China, thus implying that the healthcare system in these areas is not capable to manage T2DM efficaciously and pointing out the need for the quest for potential interventions to improve the quality of the healthcare system in rural China.

Salinas Martínez et al. investigated realistic, idealistic and unrealistic expectations for drugs (metformin, glyburide and insulin) in primary care in T2DM patients since individuals with T2DM having positive outcome expectations are prone to benefit from T2DM management as compared with those with negative outcome expectations, whereas idealistic expectations are likely to exhibit the opposite effect on health outcomes. The authors have shown that personal preferences should be taken into account when medication adherence is concerned since almost half of the patients on insulin therapy would prefer to switch to oral antihyperglycemic agents as compared to 1/4 on metformin who would like the opposite. Sex, place of residence, time since diagnosis and diabetes education were the factors significantly correlated with the expectations in this study. The reinforcement of realistic expectations is needed.

This Research Topic sums up the current information and points out the new insights into the diagnosis of insulin resistance and management of diabetes in primary care. The up-to-date findings of studies that have been published in the current Research Topic proposed some cost effective and easy available diagnostics laboratory markers for insulin-resistance management and POC testing for glycated hemoglobin, in addition to some other more expensive procedures. Also, the need for raising awareness of this metabolic disease and the necessity for the modification of behavioral risk factors and potential interventions to ameliorate the quality of the healthcare system, especially in rural regions is of paramount importance. Additional studies are needed in the future focusing on raising awareness to beneficial health behaviors, as well to find the cost effective and easy available approaches for the diagnosis and therapeutic applications of T2DM.

## Author contributions

AK and I-ST wrote the draft, reviewed, and revised the manuscript. All authors listed have made substantial, direct, and intellectual contributions to the work and approved it for publication.
